# Quantitative determination of cut efficiency during soft tissue surgery using diode lasers in the wavelength range between 400 and 1500 nm

**DOI:** 10.1007/s10103-020-03243-4

**Published:** 2021-01-26

**Authors:** Amelie Hanke, Rolf Fimmers, Matthias Frentzen, Jörg Meister

**Affiliations:** 1 Department of Operative and Preventive Dentistry, Bonn University, Welschnonnenstrasse 17, 53111 Bonn, Germany; 2 Institute for Medical Biometry, Informatics and Epidemiology (IMBIE), Bonn University, Sigmund-Freud-Strasse 25, 53105 Bonn, Germany; 3 Center of Dento-Maxillo-Facial Medicine, Bonn University, Welschnonnenstrasse 17, 53111 Bonn, Germany; 4 Center of Applied Medical Laser Research and Biomedical Optics (AMLaReBO), Bonn University, Welschnonnenstrasse 17, 53111 Bonn, Germany; 5 Laser Application in Medical Sciences Research Center, Shahid Beheshti University of Medical Sciences, Velenjak Street, 19857-17443 Tehran, Iran

**Keywords:** Efficiency factor, Evaluation masks, Histology, Interaction zones, Porcine gingiva

## Abstract

Within the scope of this ex vivo study, the cut efficiency was investigated with eight diode laser wavelengths in the range from 400 to 1500 nm. Incisions on porcine gingiva samples were generated in CW-mode at a power range of 0.5–4 W using a bare fiber (*∅* = 320 μm) in contact and non-contact mode at a cut speed of 2 mm/s. Cut depths, cut widths, and thermal damages were recorded based on histological sections and were evaluated via measurement masks. Moreover, with respect to the controllability of a therapeutic measure, an efficiency factor was defined. At powers above 2 W, for 445 nm, the maximum cut depth was 820 μm and 344 μm for 810 nm, respectively. At all wavelength and power ranges, the cut width averaged 125 μm. At minimum output power (0.5 W), the spatial expansion of the thermal damage in the tissue surface layer corresponds in the blue/green wavelength range from the very beginning of the laser impact to the fiber core diameter. It could be shown that increases in the diode laser power output do not correlate to the same extent with the incision depth nor with thermal damage to tissue.

## Introduction

Since the beginning of this millennium, diode lasers have become established as new photonic technologies in modern dentistry. Emitting light in the near-infrared (NIR) spectral region of 800–1000 nm, the applied diode lasers have expanded the range of therapy beyond conventional therapeutic measures. Their field of application extends from endodontics [1, 2] to periodontology [3, 4] down to soft-tissue surgery [5, 6]. Due to the properties of laser radiation, such as adaptable energy and power densities and monochromatism, this technology also allows carrying out specific therapeutic measures leading to various effects, e.g., accelerated wound healing, pain alleviation, and the removal of oral soft tissue without hardly any bleeding with simultaneous coagulation and thermal disinfection of the wound edges [7, 8].

In general, the interaction with tissue is based on the absorption of the radiation in the corresponding chromophores. In the wavelength region of 800 to 1000 nm, the absorption occurs primarily in melanin and hemoglobin (*μ*_A(Melanin/Skin)_ = 134–61.7 cm^−1^, *μ*_A(Hemoglobin)_ = 4.1–1.1 cm^−1^) [9, 10]. The absorption in water is hereby negligible (*μ*_A(Water)_ = 0.02–0.36 cm^−1^) [11]. With the introduction of the diode laser wavelength 445 nm in modern dentistry, new application opportunities have become accessible resulting from increased absorption values in the corresponding chromophores (*μ*_A(Melanin/Skin)_ = 1033 cm^−1^ and *μ*_A(Hemoglobin)_ = 1404 cm^−1^, μ_A(Water)_ = 2.8·10^−4^ cm^−1^) [9–11].

It is particularly interesting to medically classify the effects of diode laser emissions at the wavelengths of 445 nm, 514 nm, or also 532 nm compared to those of established wavelengths. The expert literature search, however, poses a general problem: It is difficult or even impossible to search for existing, valid comparative data and fixed reference points with respect to laser parameters such as wavelength, operation mode, and power settings as well as handling (fiber guiding) and uniform tissue parameters [12]. Table 1 illustrates this problem based on a literature research for the wavelength 980 nm.

**Table 1:** Illustration of the problems in searching for valid comparative data and fixed reference points of diode laser applications in soft tissue surgery [13–24]. Here, both the wavelength 980 nm and continuous wave (CW) laser operation mode are kept as fixed reference points. The varying fiber diameters (200–400 μm), power settings (0.5–10 W), and the applied tissue types, alone, make it impossible to directly compare the studies. Research was conducted on PubMed, Web of Science, and Google Scholar. The keywords selected were as follows: diode laser (980 nm), gingiva, and soft tissue (surgery)

Reference	Current study	[13]	[14]	[15]	[16]	[17]	[18]	[19]	[20, 21]	[22]	[23]	[24]
Study type	Ex vivo	In vitro	Ex vivo	Ex vivo	Case series	Clinical study	Ex vivo	In vivo	Case series	Case series	Case series	In vitro
Wavelength (nm)	980	980	980	975	980	980	980	980	980	980	980	980
Operation mode	CW	CW	CW	CW	CW	CW	CW	CW	CW	CW	CW	CW
Power range (W)	0.5–4	10	2/4	3/6	1.5	1.5	2.5	2	3	5	2–8	2/4
Power check (periodically)	Yes	n.a.	Yes	n.a.	n.a.	n.a.	n.a.	n.a.	n.a.	n.a.	n.a.	n.a.
Fiber type, diameter (μm)	Bare fiber, 320	Bare fiber, 320	Bare fiber, 320	Bare fiber, 320	Bare fiber, 400	Bare fiber, 320	Bare fiber, 200	Bare fiber, 400	Bare fiber, 300	Bare fiber, 320	Bare fiber, 200–400	Bare fiber, 300
Fiber initialization	Yes	Yes/no	n.a.	Yes	Yes	n.a.	Yes	Yes	Yes/no	n.a.	Yes/no	n.a.
Fiber contact pressure (Pa) or	6.7·10^−2^		n.a.	n.a.	n.a	n.a.	n.a.	n.a.	n.a.	n.a.	n.a.	n.a.
Tracking force (N)	3.5·10^−3^	4.5·10^−3^	n.a	n.a	n.a	n.a	n.a	n.a	n.a	n.a	n.a	n.a
Treatment mode	Incision	Incision	Incision	Incision	Incision	Incision	Incision	Incision	Incision	Incision	Incision	Ablation
Cutting speed (mm/s)	2	No movement	5	n.a.	n.a.	n.a.	n.a.	n.a.	n.a.	n.a.	n.a.	n.a.
Tissue samples	Pig gingiva	Chicken meat	Beef tongues	Chicken breast	Human gingiva	Human buccal mucosa	Pig mandible	Rabbit tongues	Human lip	Human tongues	Human buccal mucosa	Pig gingiva

*n.a.* not available

Consequently, within the scope of this study, the effects of diode lasers equipped with various modules operated at eight different wavelengths in the spectral region of between 400 and 1500 nm are compared with one another under identical and reproducible ex vivo conditions. This study primarily investigates the cutting behavior, especially the determination of the cut depths and widths as well as the thermal effects in the surrounding areas of the incisions based on histological evaluation. As a result, the cut efficiency in the form of an efficiency factor is calculated, and its applicability is verified by comparing it with the literature.

According to the null hypothesis, it is assumed that, at all wavelengths, there is a continuous increase in the cut efficiency, an increase in incision depth and in thermal tissue damage at increasing laser output powers.

## Materials and methods

### Laser sources

For this study, two different laser devices (Fox® and Wolf®, A.R.C. Laser GmbH, Nuremberg, Germany) were used. For this investigation, both devices were equipped with various diode laser modules. Table 2 presents the distribution of the wavelengths between the corresponding devices.

**Table 2:** Diode laser device types Fox® and Wolf® equipped with the corresponding wavelength modules

Device types	Fox®	Wolf®
Laser modules (wavelength)	514 nm	405 nm
	810 nm	445 nm
	980 nm	532 nm
	1064 nm	1470 nm

**Fig. 1:**
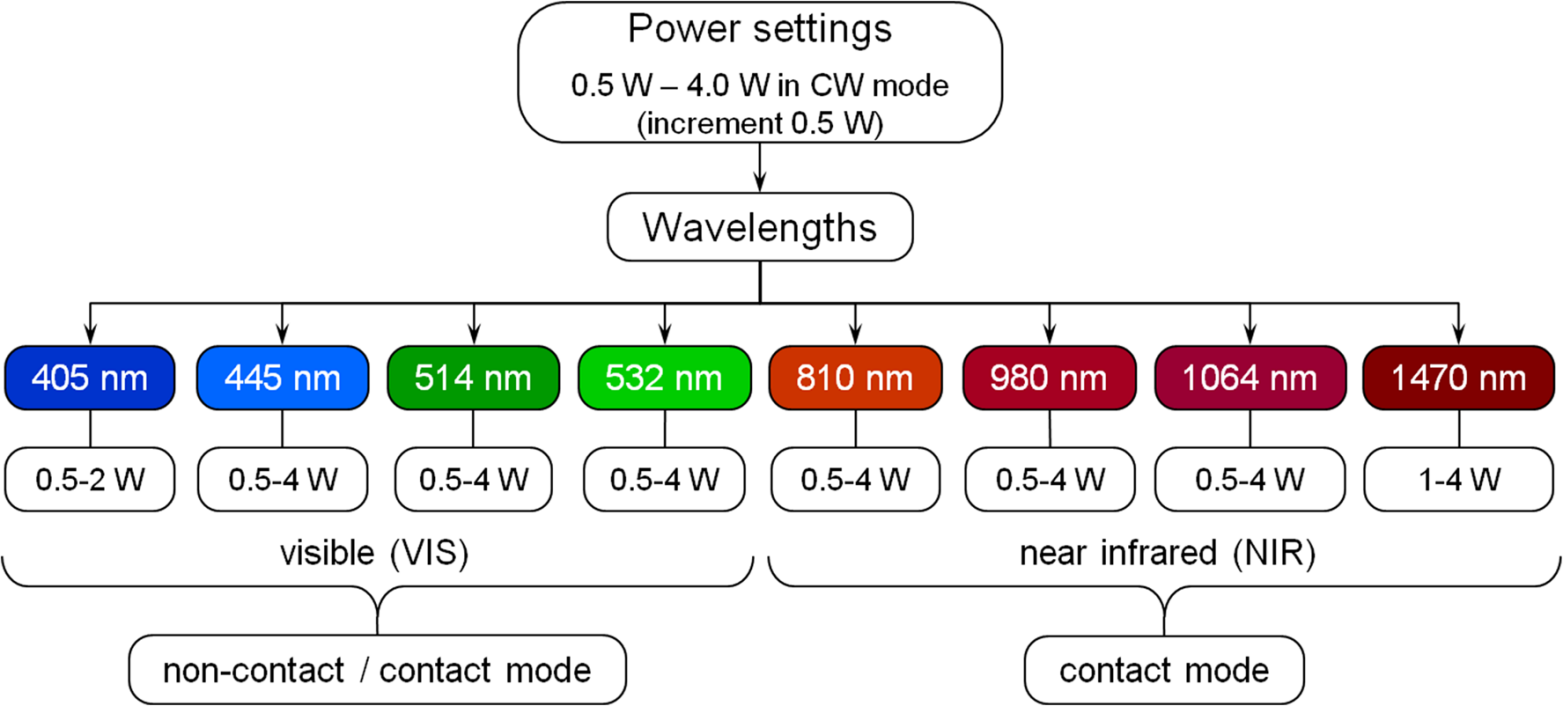
Overview of module wavelengths and the applied power range. For generating the incisions, powers of 0.5 W to 4 W were applied in 0.5 W- increments. Limitations in the power range resulted at wavelengths 405 nm (max. 2 W) and 1470 nm (min. 1 W). Light of wavelengths in the VIS-range was used for experiments in both the contact and non-contact mode. However, at wavelengths in the NIR-range, only the contact mode was applied

All the incisions were conducted in continuous wave (CW) mode. The indication-based power range was 0.5 W to 4 W at increments of 0.5 W. Two laser modules showed limitations in the power range: at a wavelength 405 nm, a maximum power output of only 2 W could be reached; at 1470 nm, the minimum power output was 1 W (Fig. 1).

In the spectral region 405–532 nm, the irradiations were conducted in contact mode and non-contact mode. Only the contact mode was applied for the wavelengths of ≥ 810 nm due to the negligible absorption in the epithelium of gingival soft tissue and the reduced absorption in the hemoglobin of the subepithelial connective tissue [25]. Under clinical conditions, incisions in the non-contact mode will not be performed in this wavelength range and the laser settings used.

The laser radiation for both device types was guided to the tissue sample via a “bare” fiber (*∅*_outer diameter_ = 320 μm and *∅*_core diameter_ = 280 μm). The distal end of the fiber was fixed by using an application handpiece with a metallic guiding sleeve. The fiber protruded 20 mm from the guiding sleeve to have high flexibility.

### Sample preparation and sample number

Gingiva samples from freshly slaughtered pigs (German landrace, 8 weeks old, mass 30–40 kg) were used for laser irradiation. Samples from lower pig jaws (porcine models) were extracted with a band saw (MICRO-Band saw MBS 240/E, Proxxon S.A., Wecker, Luxemburg) within 1 h postmortem. Five block resections per lower jaw were taken, whereby each sample was 2 cm long and approx. 1.5 cm high. Until the irradiation was carried out, the samples were covered with gauze saturated with a sterile rinsing solution (0.9% physiological saline solution, 0.01‰ sodium azide, Merck, Darmstadt, Germany) and stored cooled at 4 °C.

The number of samples depended on the power output parameter set (0.5–4 W) and on the handling (contact and non-contact mode). Two block resections were earmarked (4–5 cuts per resection) for each power output parameter set. In the contact mode, the irradiation took place at all 8 wavelengths: thus, with 5-fold repetition, this resulted in a sample number of 80 block resections. In the non-contact mode, the sample number was 40 block resections due to the unfeasibility caused by negligible absorption in the epithelium and the reduced absorption in the hemoglobin of the subepithelial connective tissue of radiation in the near-IR range: this means that this part of the study was conducted only with the lasers in the visible spectral region.

### Experimental setup

**Fig. 2:**
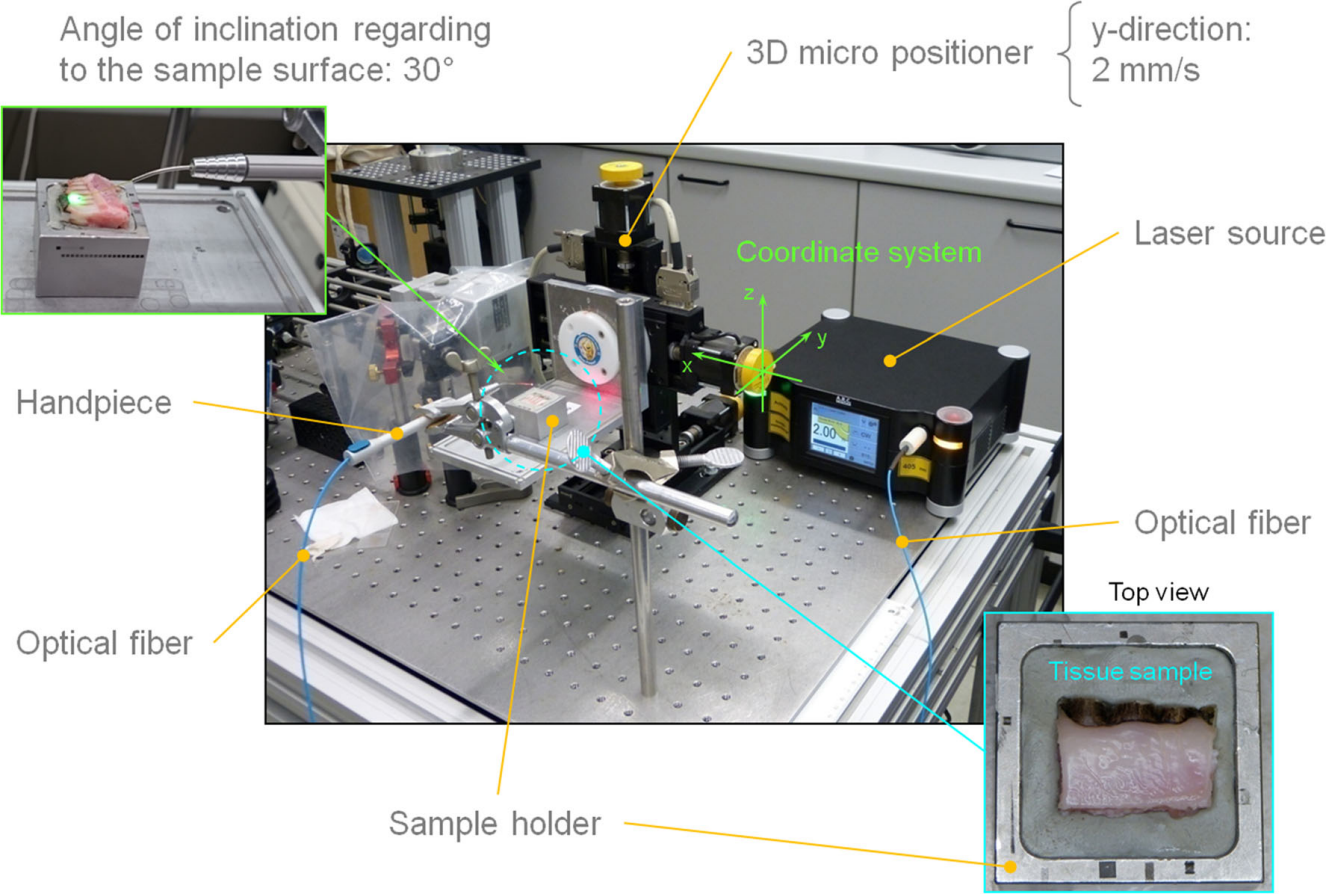
Experimental setup: here with the Wolf-system (laser source) on the optical bench. The sample holder (bottom right) is fixed on a 3D computer-controlled micropositioner which moves the sample at a speed of 2 mm/s along the y-axis. The laser radiation is transmitted to the sample via an optical fiber. The positioning of the optical fiber over the sample at an inclination angle of 30° was realized using a formable guiding sleeve (bendable) connected to an application handpiece (top left). The length of the fiber projection was 20 mm

With the target of ensuring maximum reproducibility, all influenceable mechanical and optical variables such as cut direction, cut speed, cut distances, fiber distance to sample surface (contact or non-contact), fiber contact pressure (initial condition), and control of the laser power were standardized as far as possible. Figure 2 shows the experiment setup.

The block resections were embedded in a magnet holder, filled with a plastic silicone mass (Contrast putty soft, VOCO GmbH, Cuxhaven, Germany) (Fig. 2, bottom right). In turn, the magnet holder was located on a 3D computer-controlled micropositioner (VT-80, Micos, Eschbach, Germany), enabling the positioning with a precision of 1 μm.

For every block resection, the start and end positions were determined by means of software calibration. During irradiation, the sample was moved constantly at a speed of 2 mm/s along the y-axis. The handpiece with the metallic guiding sleeve and integrated fiber was located in a fixed position above the sample, whereby the guiding sleeve showed an inclination angle of 30° to the sample surface (Fig. 2, top left). The sample movement itself was in a parallel orientation to the plane of the inclination angle.

**Fig. 3:**
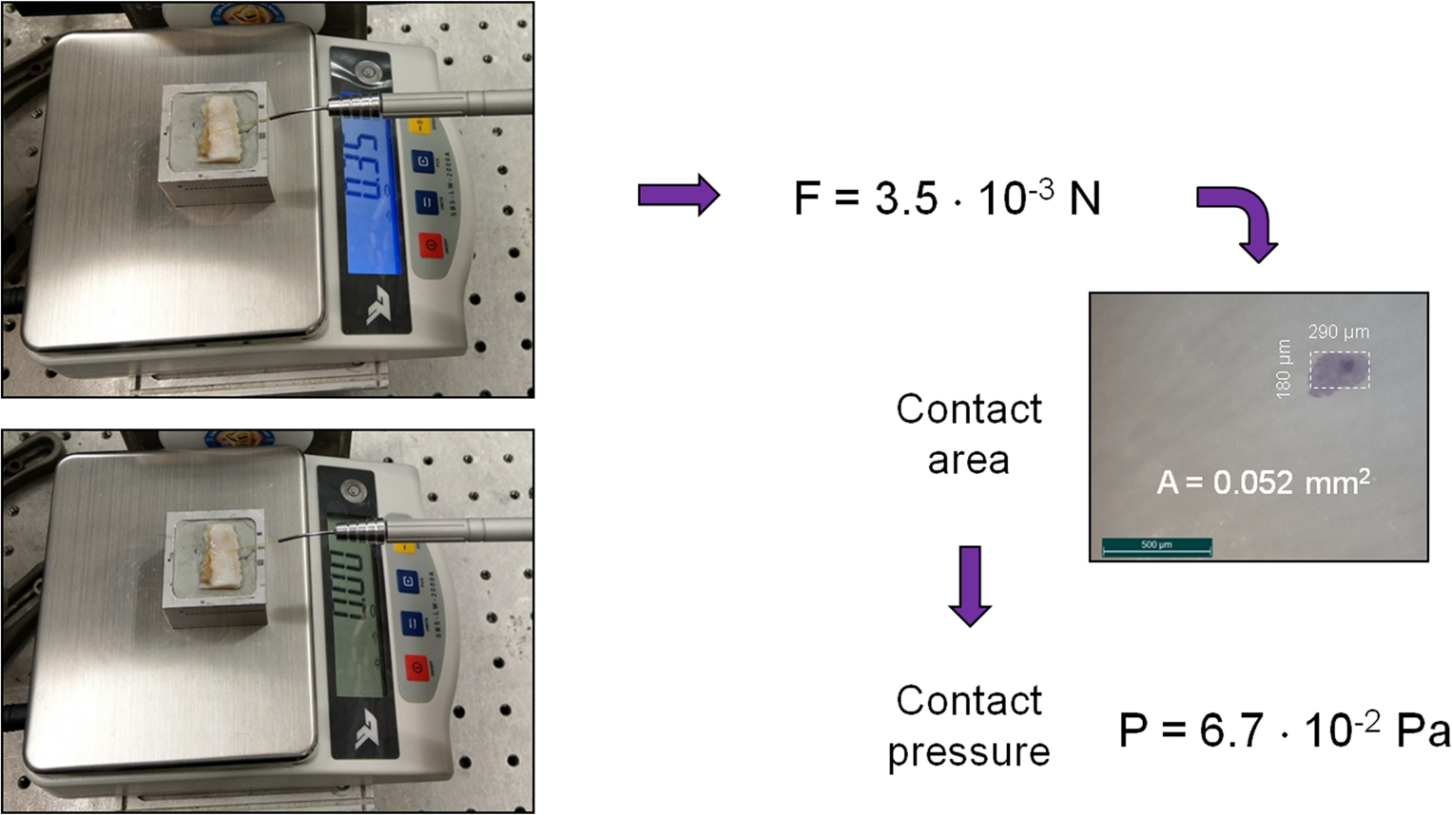
Measured contact pressure of the fiber on the tissue surface by using an electronic scale. For determining the contract pressure, the contact area was determined using a color imprint of the fiber tip on the tissue surface

The contact pressure on the sample was checked in regular intervals (after irradiation of 4 to 5 block resections; Fig. 3). The contact pressure was calculated at 5.7–7.7·10^−2^ Pa as a standard, after determining the track force (3–4·10^−3^ N) via an electronic scale (SBS-LW-2000A, Steinberg Systems, Zielona Góra, Poland) and the contact area (~ 0.052 mm^2^) by means of a color imprint on the sample surface.

Before every individual cut, the emitted laser power output was measured at the distal end of the fiber via an energy/power meter (LabMax Top, Coherent, Santa Clara, CA, USA) combined with a PM10 detector (Coherent, Santa Clara, CA, USA). Output deviations of ≥ 5% necessitated a new configuration of the fiber tip.

### Irradiation procedure

The irradiation of the samples took place on the same day of the slaughter. Before irradiation samples were removed from the fridge to obtain room (lab) temperature (~ 24 °C). A specially designed protocol (checklist and photo documentation) ensured a standardized experimental procedure. During the irradiation, the gingival soft tissue was left on the bone substance which ensured an almost smooth gingival surface. For all irradiation procedures in contact mode, a marking line was set at an angle of 90° relative to the direction of the incision, which, moreover, guaranteed an immediate interaction with the tissue at the irradiation begin (initialization) [15]. This kind of initialization corresponds to a carbon-based tissue layer at the distal end of the fiber based on irradiation of an organic matrix [26]. Depending on the morphological state of the block resection, 4–5 cuts were generated 4 mm apart on the gingival surface. In the non-contact mode, the fiber end was directed in a distance of 0.5 mm over the gingiva surface. The protocol was identical to that for contact mode with the exception of the initialization which was not conducted in non-contact mode.

After the irradiations, the gingiva specimens were detached from the bone and stored in 4%-formaldehyde solution (Merck, Darmstadt, Germany).

### Histological preparation

The gingival specimens were stored in the formaldehyde solution at 4 °C for 24 h. This procedure minimized tissue shrinkage because the swelling and subsequent formol fixation almost balanced one another [27]. The dehydration using an alcohol series (30%, 50%, 70%, 80%, 90%, 95%, and 100%) and the embedding in paraffin occurred in an automated process (Tissue-Tek VIP 2000, Model 4622, Miles Scientific, Napierville, IL, USA). After dehydration, the gingival specimens were embedded in paraffin blocks (Tissue-Tek, Modell 4715, Sakura Finetek Europe, Zoeterwoude, Netherlands). Histological sections of 2 to 4 μm thickness were produced on a Section Transfer System (HM 355 S, Microm Int. Walldorf, Germany). After deparaffinization, the sections were stained with hematoxylin and eosin (HE) for the subsequent histological evaluation.

### Histological evaluation

**Fig. 4:**
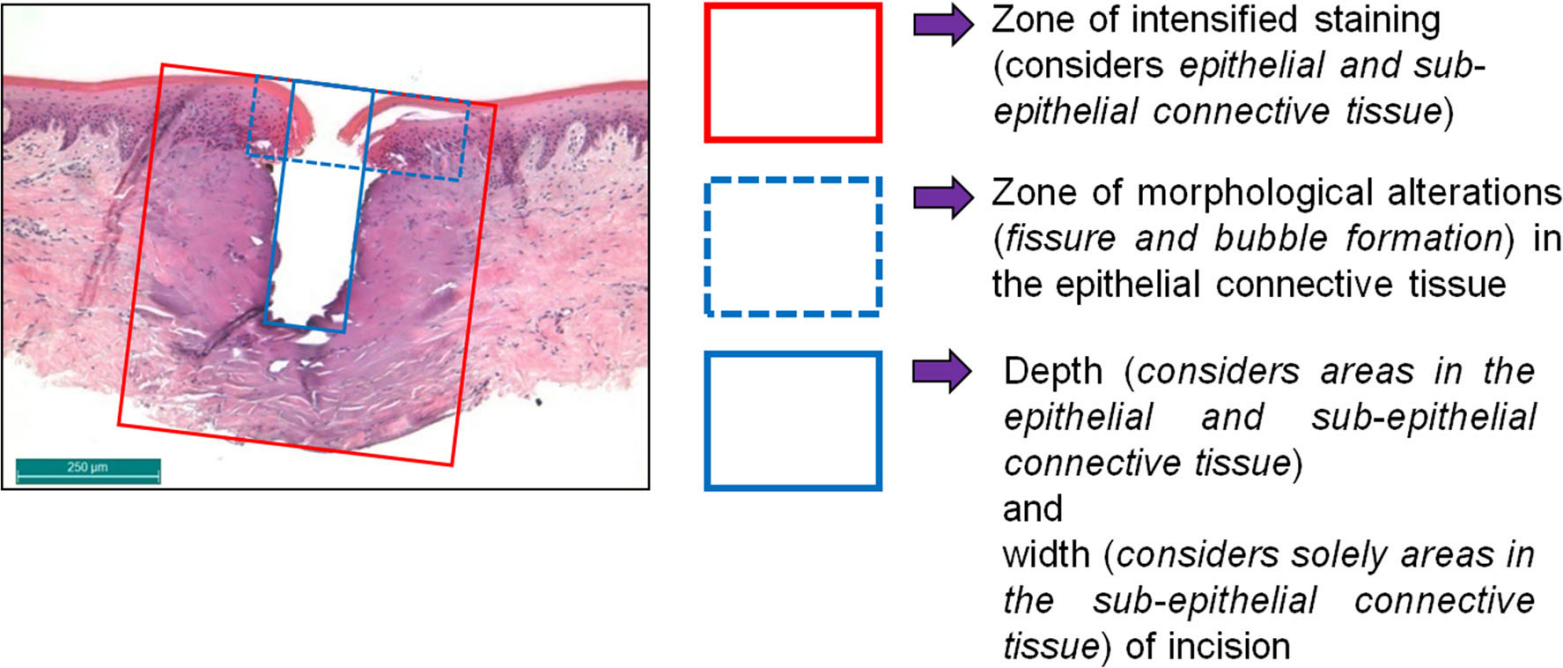
Measurement masks for determining the dimensions of visible changes in tissue. Incision depths and widths as well as zones of thermal and morphological damage were recorded geometrically. The colored lines depict the following: blue solid line, incision depth and width; blue dashed line, zones of morphological changes; and red line, maximum expansion in width and depth of the areas of intensive staining through the effect of temperature (defined as Total Interaction Zone, TIZ)

Respectively one histological specimen, on which all laser incisions of a block resection were evaluable, was used for the histometric evaluation employing a transmitted light microscope (DM 1000, Leica Mikrosysteme, Wetzlar, Germany). The simultaneous photo-documentation took place via a microscope camera (DFC 420 C, Leica Microsystems and the corresponding Software LAS – Leica Applications Suite V3.8). To characterize the different areas of visible changes, measurement masks were created for defining the depth and width of the incisions as well as the zones of thermal and morphological damage. These masks were placed tangentially to a common cranial boundary on which morphologically unchanged edges of gingival epithelium lies (Fig. 4).

**Fig. 5:**
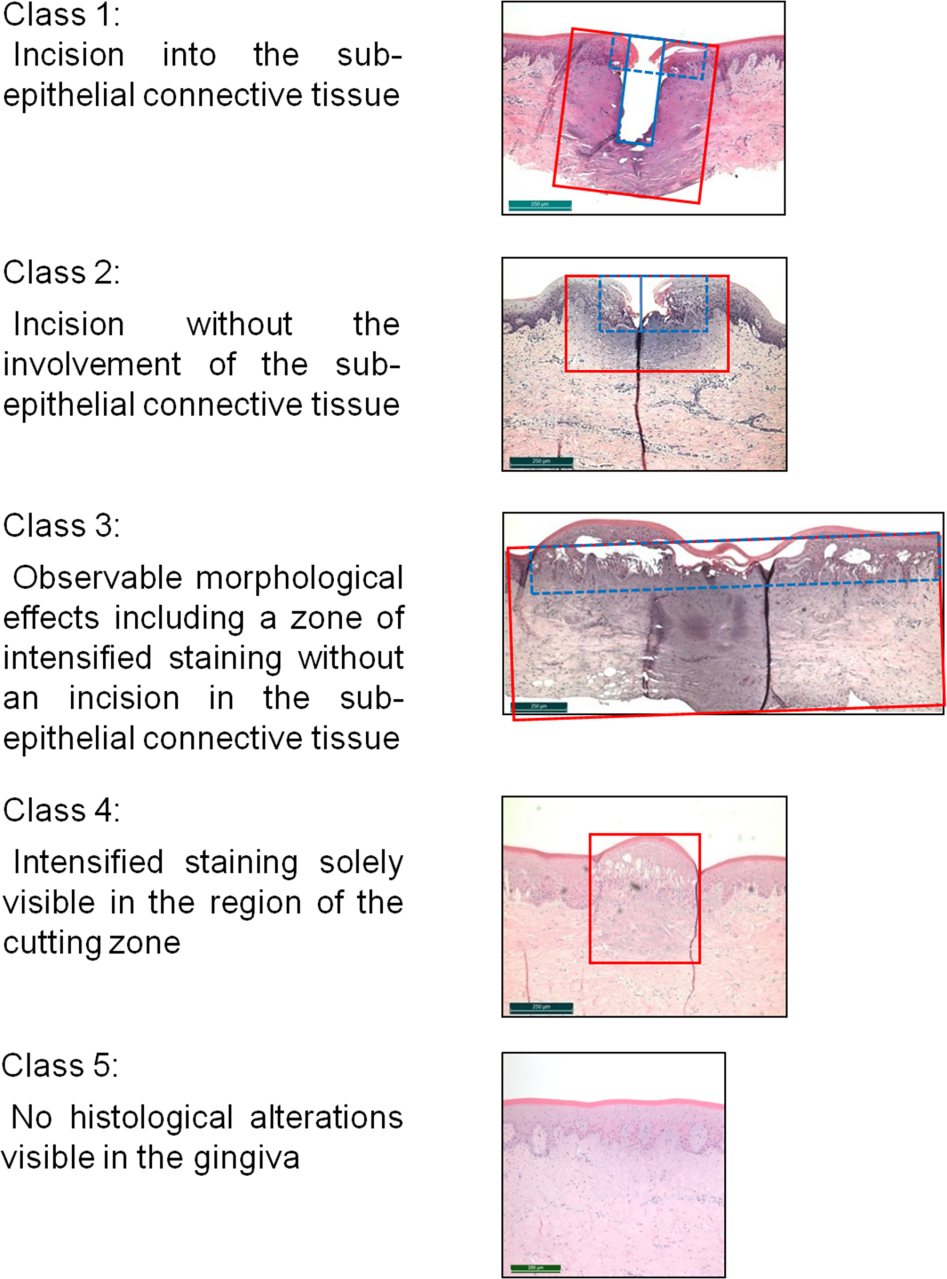
Allocation of incisions to classes 1 to 5 based on the measurement masks

On the basis of the measurement masks, the various manifestations were classified to five classes. Here, the classification criteria were oriented to the presence of a therapeutically relevant incision effect (Fig. 5).

### Definition: efficiency vector $\overset{\rightharpoonup }{\upgamma}$

The efficiency vector $\overset{\rightharpoonup }{\gamma }$ is a newly defined parameter for quantifying the laser-tissue interaction. This vector describes the momentary states of tissue during the irradiation (laser properties) at any site in the tissue (tissue properties). This results in the following equation (Eq. 1):

1 $$ \overset{\rightharpoonup }{\upgamma}=\overset{\rightharpoonup }{\upgamma}\left(\mathrm{laser}\ \mathrm{properties},\mathrm{tissue}\ \mathrm{properties}\right) $$

whereby the laser properties are the wavelength, output power, intensity, operation mode, irradiation time, and beam profile. Tissue properties are, e.g., the tissue composition, absorption and scattering (reduced scattering) coefficient, anisotropy factor, perfusion, thermal conductivity, and specific heat capacity.

By maintaining the external conditions (sample preparation, experimental setup, and irradiation procedure), additionally favored by the use of a “bare”-fiber with frontally oriented emission characteristics, the efficiency vector $\overset{\rightharpoonup }{\gamma }$ can be reduced to one dimension to *γ*_z_ (efficiency factor for determining the depth effect of the radiation). During the laser application, the depth effect can be hereby subdivided into mechanical effects (cutting depth *d*_cut_) and thermal effects (coagulated tissue, zones of intensified staining). The efficiency factor *γ*_z_ now enables quantification of the energy distributions in tissue. Equation 2 elucidates this relation as follows:

2 $$ {\gamma}_z=\frac{\mid \mathrm{cuttingdepth}\left({d}_{\mathrm{cut}}\right)\mid }{\mid \mathrm{totalinteractionzone}\left(\mathrm{TIZ}\right)\mid } $$

The term “total interaction zone” (TIZ) hereby denotes the maximum expansion of intensified staining into the depth includes the cutting depth (*d*_cut_) plus the zone of thermal damage in z-dimension of the tissue (Fig. 4). The efficiency factor *γ*_z_ can assume values of between 0 and 1 (or 0% and 100%), whereby the value 1 (|TIZ| = |*d*_cut_|) corresponds to a cut without thermal damage, i.e., a scalpel incision.

Therefore, the TIZ defines the tissue sector that is touched by the intervention. Due to healing and inflammatory processes, it should be kept as small as possible.

### Statistical evaluation

Histological based data of cut depth, cut width, and thermal damage zones were determined with the microscope camera DFC 420 C (Leica Microsystems and the corresponding Software LAS, Leica Applications Suite V3.8). Descriptive statistics, including calculation of the mean values, standard deviations, and an error estimation, were applied to the cut depth, TIZ in depth and width, and for *γ*_z_ using the spreadsheet calculation program OriginPro 8G (OriginLab Corporation, Northampton, MA, USA). Graphical evaluation of the results was carried out as well with OriginPro 8G. Splines and linear fit functions were used for visualization of the data behaviors.

Explorative statistical analysis of *γ*_z_ was based on a linear model with laser output power as covariate and wavelength as a factor. If a clear evidence for interaction is determined, sub-analyses will be performed separately for the wavelength, fitting linear regressions with power as a covariate, and for the power by an analysis of variance followed by Tukey’s test for pairwise comparisons of the wavelengths.

## Results

### Histological overview

**Fig. 6:**
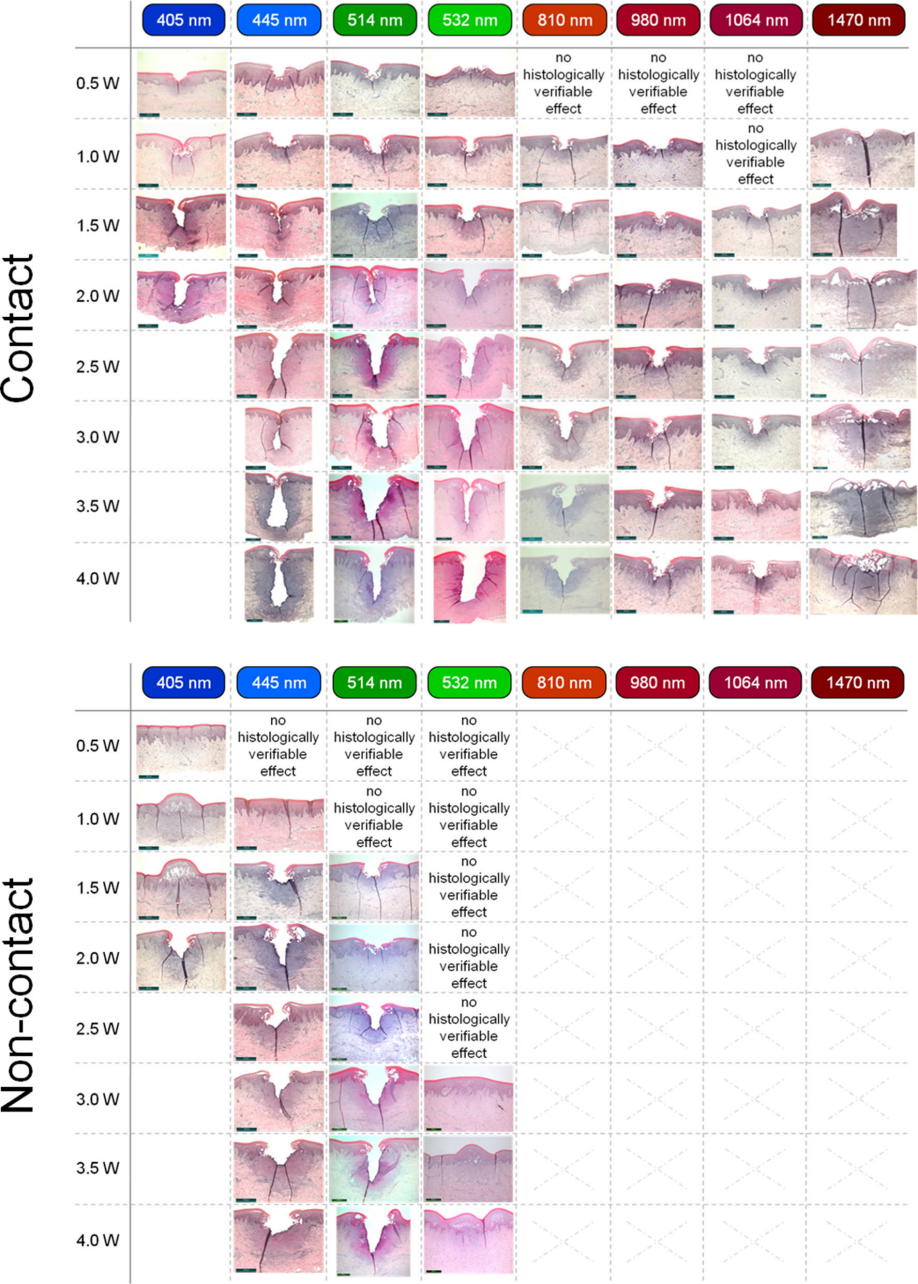
Overview of histological sections from diode laser operation in contact mode (above) and non-contact mode (below) depending on the applied wavelengths and power outputs. Due to the low absorption of light in the NIR wavelength region (810–1470 nm) in gingival soft tissue, only the wavelengths in the visual region (405–532 nm) were used for operation in the non-contact mode

Altogether, 437 incisions from 120 jaw resections were histologically recorded, measured, and classified. A class-typical incision image resulted for every combination of factors consisting of wavelength, laser power output, and irradiation mode (contact and non-contact; Fig. 6).

Parameter-specific threshold values for class ≤ 2 incisions (Fig. 5) could be determined for the diode laser modules operated at all wavelengths and power settings. For the contact mode, these values were 0.5 W for the visible spectral range (405–532 nm) and 1 W for the NIR-wavelengths 810 nm and 980 nm. However, at the wavelengths 1064 nm and 1470 nm, incisions could be observed only as of 1.5 W and 4 W, respectively. In the non-contact mode, the threshold value at wavelength 405 nm was 2 W; at 445 nm and 514 nm, this threshold value was 1.5 W. However, no incision effect was observed at a wavelength of 532 nm. This behavior clearly points to the trend of reduced tissue interactions at longer wavelengths (≥ 532 nm). Only thermal changes in the epithelial tissue could still be histologically proven at 532 nm and power outputs of ≥ 3 W.

### Cut depth and width

**Fig. 7:**
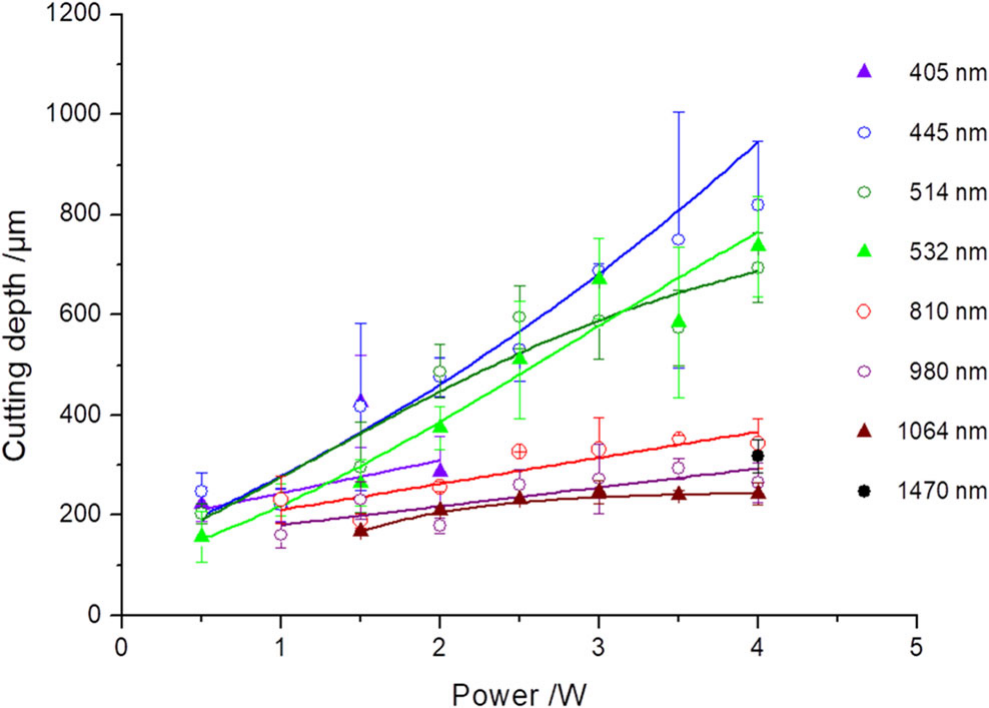
Cut depths for incisions of classes of ≤ 2 determined as a function of the applied power output. The results found at wavelengths of the visible region of 445 nm, 514 nm, and 532 nm show a considerable increase in cut depth in the power range of ≥ 2 W compared to those determined at NIR-wavelengths 810 nm, 980 nm, and 1064 nm

Figure 7 shows the cutting behavior of the diode lasers operated at various wavelengths and applied power output. A steady increase in the cut depth correlates with the rise in the power output. In the power range of up to 1 W, all cut depths lay at a comparable order of magnitude of 214 μm ± 31 μm. Above 2 W, however, clear differences emerged among cut depths found at wavelengths in the visible spectral range (445 nm, 514 nm, and 532 nm) and those in the near-infrared range (810 nm, 980 nm, and 1064 nm). Hereby, a maximum cut depth of 820 μm was found at 445 nm, and that of 344 μm at a wavelength of 810 nm. The mean descriptive observable statistical fluctuation range is in the order of ± 16% for the visible laser lines and ± 10% in the NIR.

A cut width of 125 μm ± 24 μm (averaged over all wavelengths and all power output ranges) was determined for all class 1 incisions. Thus, all recorded cut widths were below the core diameter of the optical fiber (*∅*_core diameter_ = 280 μm).

### Thermal effects

**Fig. 8:**
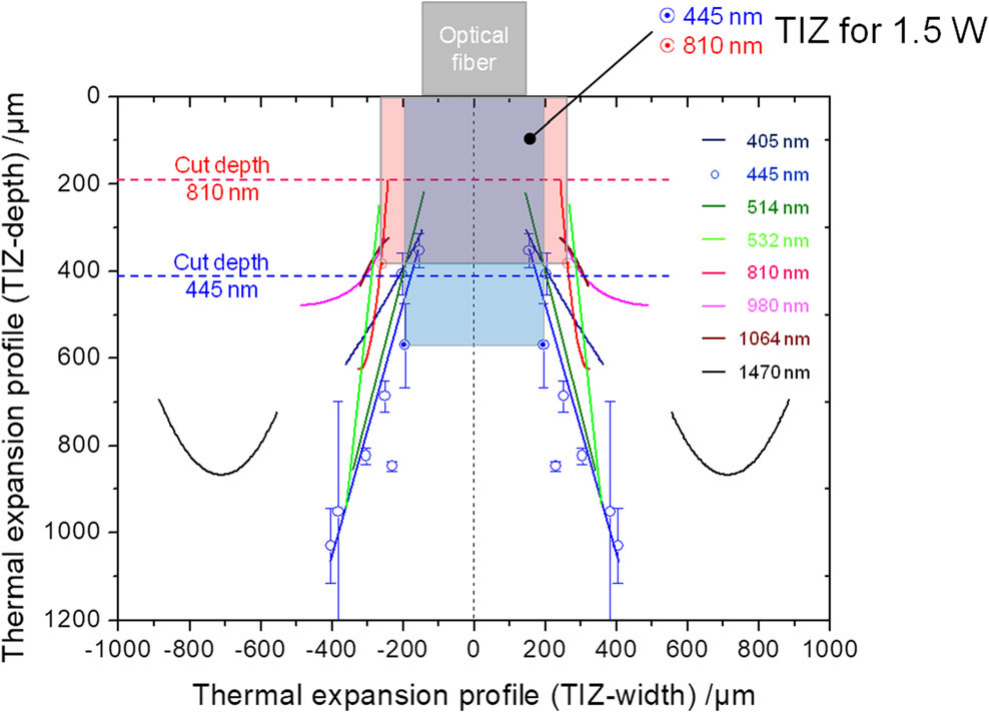
Thermal expansion profiles (TIZ in width (x-axis) and depth (y-axis) for incisions class ≤ 2) in gingival soft tissue presented for all wavelengths in contact mode (compare Fig. 6). As an example, data points and error bars are shown for the wavelength 445 nm. Every data point (pairwise + and −) corresponds to a clearly defined power setting (0.5–4 W in 0.5 W increments). At a power output of 1.5 W, noteworthy, here are the dimensions of the thermal damage zone found at the wavelengths 810 nm (wide and flat red zone above) and 445 nm (narrower deep blue zone above). Additionally, the respective cutting depths are delineated for these wavelengths. Here, the ratio (*γ*_z_, efficiency factor) of cutting depth (*d*_cut_) to the entire thermal depth expansion (TIZ) is decisive for determining the cut efficiency

Figure 8 presents the thermal expansion profile in the gingival tissue as a function of the wavelengths. Noteworthy, here are the heterogeneous width dimensions for the wavelengths in the near-infrared region (NIR, 980 to 1470 nm). Unlike those results, the results at 445 nm and 514 nm show a uniform thermal effect zone with increasing power. This could also be observed at the wavelengths 532 nm and 810 nm, whereby the thermal effect zone was altogether widened in the lower power range; this confirms the trend to an expanded thermal effect at longer wavelengths. However, one exception is the wavelength 405 nm, whose thermal expansion profile in the lower power range is comparable to those found at 445 nm and 514 nm and whose dimensions obtained at 2 W are similar to those observed at 532 nm and 810 nm.

At the wavelengths 405 nm, 445 nm, and 514 nm, the thermal damage starts to show almost the same dimensions corresponding to the core diameter of the optical fiber (*∅*_core diameter_ = 280 μm). At the wavelengths ≥ 532 nm, the damage zones are widened from the very beginning. An error estimation (mean descriptive observable statistical fluctuation range) in depth of the TIZ shows slight differences between the visible (± 12.5%) and the NIR lasers (± 9%) whereas in width, the gap of the mean fluctuation range of the TIZ between visible wavelengths (± 18.5%) and the NIR (± 13%) is increased (not shown in Fig. 8).

### Results of efficiency

**Fig. 9:**
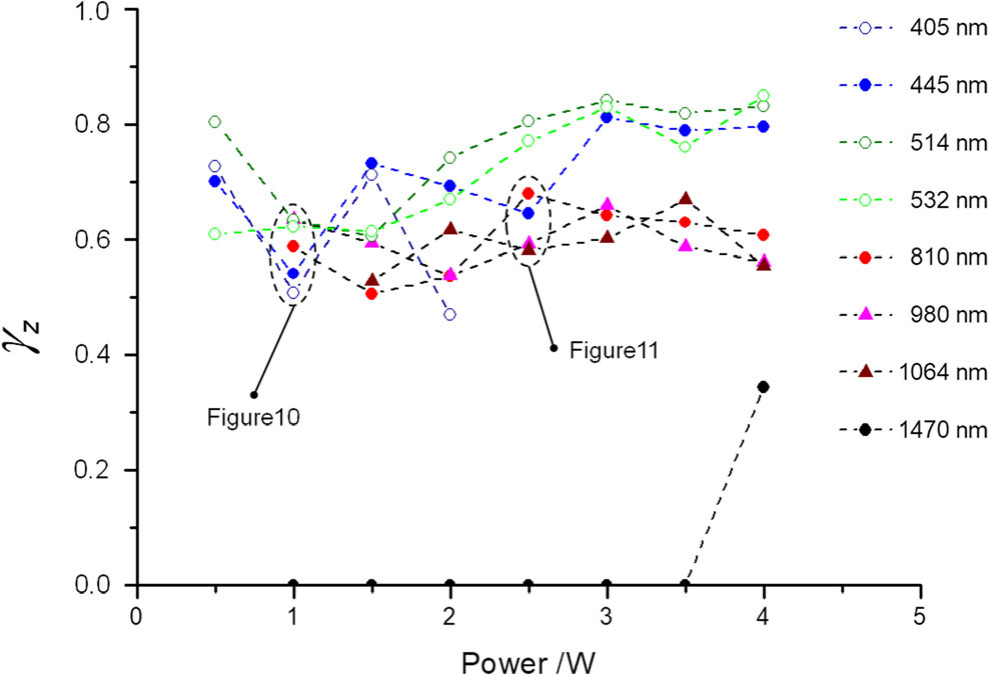
Efficiency factor *γ*_z_ for the wavelengths 405 to 1470 nm vs. the applied power output range of 0.5–4 W. Between 1 and 2.5 W, all the diode laser wavelengths relevant for dentistry (445 nm, 810 nm, 980 nm) show a similar cut efficiency. However, as of 2.5 W, the cut efficiency of the diode laser modules in the visible spectral range predominates. To directly compare the wavelength results, now regions were selected where the cut efficiencies are relatively close to one another (1 W and 2.5 W), which means where the thermal damage zones are comparable, i.e., (dashed ovals). As Figs. 10 and 11 show, the histological sections can be directly compared with one another. Moreover, now conclusions can be drawn how the light emissions at the particular wavelengths explicitly affect the cutting behavior

For elucidating the importance of the efficiency factor *γ*_z_, in Fig. 8, the thermal damage zones were delineated for 445 nm (blue) and 810 nm (NIR) as well as the corresponding cut depths for the laser power output 1.5 W. From the ratio of cut depth (*d*_cut_) to thermal damage (TIZ), the efficiency arises or the ratio of the cutting effect to the entire applied energy for generating an incision. This value is approx. 63% at 445 nm and approx. 50% at 810 nm. This results in a wavelength- and power output-dependent efficiency behavior. Figure 9 depicts the efficiency factor γ_z_ over the entire parameter range.

This complex wavelength and power dependency of *γ*_z_ proved to be statistically significant with a *p* value of < 0.0001. Notable here is the discontinuous behavior of the efficiency at every wavelength. Depending on the power output, there is an increased jump behavior for the blue and for the NIR-wavelengths (810 nm, 980 nm, and 1064 nm) in the power range of between 0.5 and 2 W. The efficiency is somewhat smoother at the green wavelengths 514 nm and 532 nm. Nevertheless, an analysis separated for the wavelength revealed an increase of *γ*_z_ with a power raise for 445 nm, 514, and 532 nm (*p* = 0.0011, *p* < 0.0001, and *p* < 0.0001, respectively) and no indication for a trend with increasing power for the NIR wavelengths.

**Fig. 10:**
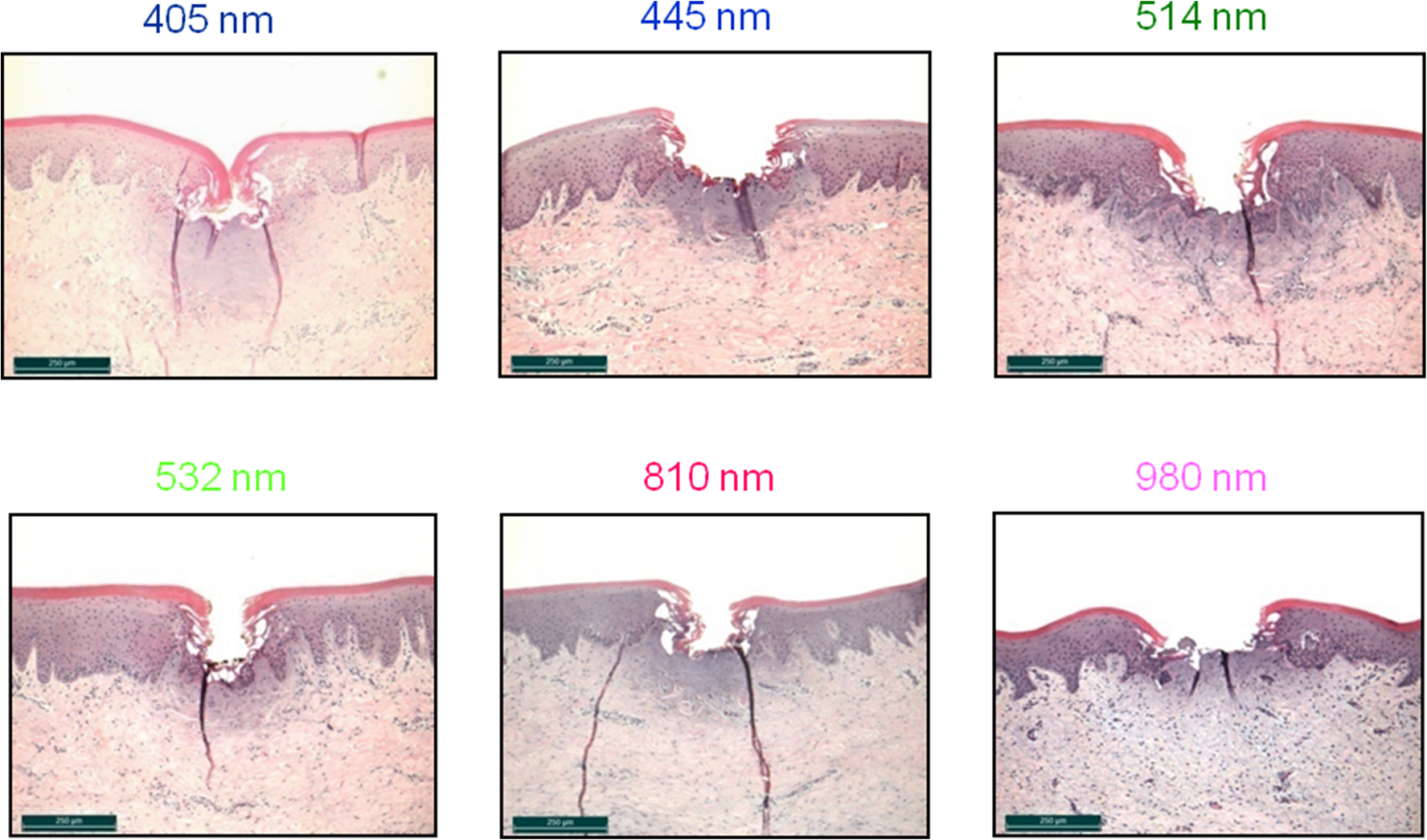
Overview of the histological sections for the wavelengths (405 to 980 nm) at a diode laser power output of 1 W. For all the wavelengths, the cut depths lie in the boundary range between the epithelium and the subepithelial connective tissue (class 2 incisions) with comparable thermal damage zones

**Fig. 11:**
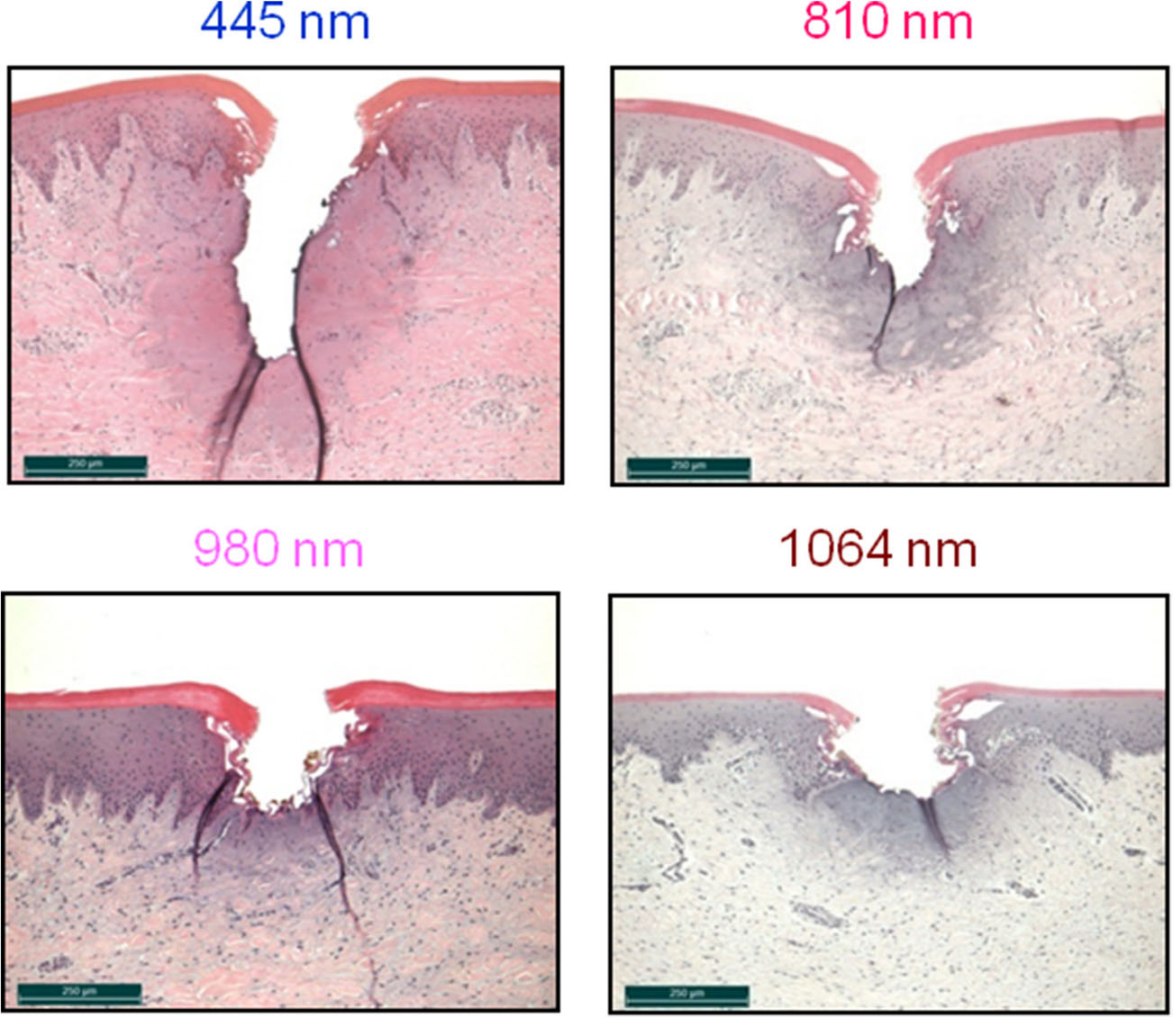
Overview of the histological sections for the diode laser wavelengths (445 nm, 810 nm, 980 nm, and 1064 nm) at a power of 2.5 W. The thermal damage zones are also comparable here. Particularly, apparent here is the effect of the diode laser emission at wavelength 445 nm regarding the applied cut depth

Moreover, there are power ranges in which the efficiency factors of various wavelengths converge, e.g., for the power range 1 W, where efficiency factors of between *γ*_z_ = 0.5 and *γ*_z_ = 0.65 can be found at wavelengths ranging from 405 to 980 nm. Despite this convergent behavior, the statistical analysis shows significant differences of the *γ*_z_ values (*p* ≤ 0.0226) between the green and the blue wavelengths (dashed oval Fig. 10). At 2.5 W (dashed oval Fig. 11), no statistical significance was found for NIR and the blue wavelength 445 nm (*p* ≥ 0.5774) among each other. For powers of > 2.5 W, there is a clear distinction between the efficiency at the wavelengths of the VIS-region (*γ*_z_ ≈ 0.8) and at the wavelengths of the NIR-region (*γ*_z_ ≈ 0.6). This could be statistically confirmed (*p* ≤ 0.009).

### Influence of the wavelength

The convergence of the efficiency factors (Fig. 9) allows a direct comparison of the tissue sections and shows the immediate effect of the diode laser emissions at particular wavelengths on the incisions along with comparable thermal damage of the surrounding tissue. Figures 10 (for 405 to 980 nm at 1 W) and 11 (for 445 nm and 810 nm to 1064 nm at 2.5 W) elucidate these circumstances.

At a power of 1 W, the maximum cut depths at all wavelengths lie in the boundary between the epithelium and the subepithelial connective tissue (class 2 incisions). At 405 nm to 810 nm, the cut depths averaged 230 μm. By contrast, at 980 nm, this value of approx. 170 μm is lower. The corresponding behavior is shown also by the total interaction zone (TIZ) value. Hereby, the respective TIZ-depths are approx. 400 μm at the wavelengths 405 to 810 nm and 250 μm for the wavelength 980 nm.

However, at a power of 2.5 W, how emissions at particular wavelengths influence the tissue interaction (class 1 incisions) becomes clearly more visible. At a comparable efficiency *γ*_z_ value of between 0.6 and 0.75, a continuous reduction in the cut depths can be seen at increasing wavelengths.

A cut depth of approx. 530 µm could be detected at a wavelength of 445 nm. With increasing wavelength from 810 → 1064 nm, the cut depth successively decreased from 330 to 260 μm and, finally, to 230 μm. Here, too, the thermal tissue effects around the incisions are comparable at all wavelengths, whereby the TIZ values vary according to the different cut depths.

## Discussion

### Valuation of the method

Pig mandibles were selected as specimens, because they greatly resemble human oral tissue in dimension and structure [28]. Although the samples were processed directly after slaughter, a direct comparison to human tissue can be made only with reservations due to the missing blood circulation. Even so, the results are valid and can be implemented in practice (the “Clinical relevance” section). Furthermore, all results are based exclusively on oral gingival tissue, which limits their transfer to other tissue types. For this, other tissue-specific studies (e.g., oral mucosa) are necessary.

The measures for standardization, reproducibility, and quality control of the experimental setup, conditions, performance, documentation, and sample preparation allowed for a maximum possible reduction of potential sources of error. Although the “Histological evaluation” section only considers a two-dimensional manifestation, through its systematics, it is nevertheless suited as a basis for comparing the dimensions of histological changes in oral soft tissue after a laser application [29, 30].

The test series were evaluated only for the contact mode. In the non-contact mode, the results were not conclusive, because the smallest irregularities and pigment differences of the gingiva are pertinent for the irradiation results. This is due to the uninfluenced light-tissue interaction process by the use of a non-initialized optical fiber. Intensity and absorption of the light on the tissue surface are strongly influenced by surface irregularities and pigmentation. This is reflected by the higher threshold values in the non-contact mode. In contrast, an initialized optical fiber in contact mode acts as a “hot tip” which leads to an immediate clinically tissue effect.

### Histological overview

Figure 6 gives an overview about the incision behavior of the modalities operated at the different wavelengths at the corresponding laser outputs in contact mode. Despite the high fraction of water in soft tissue [31], the absorption by water is negligible over the wavelength range 400 to 1100 nm. By contrast, the absorption in the chromophores melanin and hemoglobin [9, 10] lies far above the absorption of water [11], by a factor of > 10^6^ in the VIS-spectral range and by a factor of > 10^2^ in the NIR spectral range up to 1064 nm, a fact which, among others, explains the low threshold value (≤ class 2 incisions) of 0.5 W for the laser wavelength in the visible spectral region. At a wavelength of 1470 nm, however, the dominance of the water absorption is shown, a fact which, with a *μ*_A(Water)_ of 10^1^ cm^−1^, substantiates the largest thermal damage zones [11, 32].

### Cut depth and width/thermal effects

The histological results (Fig. 6) are reflected in the determined cutting performances (Fig. 7). Up to 1 W, the incisions of the laser light at wavelengths 405 to 980 nm display comparable cutting depths. As of powers of > 1 W, the cutting depths of the diode laser irradiation in the visible spectral range clearly predominate. The greater incision depths with simultaneously lower thermal side effects on oral soft tissue are confirmed by Braun et al. for the effect of blue laser light in comparison to that of wavelength 970 nm [29]. This behavioral trend could also be demonstrated by Fornaini et al. upon comparing the results for wavelengths 450 nm, 532 nm, and 808 nm, whereby the lasers working in the visible spectral range showed only slight differences with respect to temperature and cutting behavior [33]. Mergio et al. [34] studied the thermal effects on tissue of laser radiation of various wavelengths (among others, 532 nm and 808 nm) with output parameters of 2 W and 4 W or 3 W and 5 W. For all output parameters, for the surface and in depth, considerably lower temperature increases were determined in tissue for the green wavelengths than for 808 nm.

Against this literary backdrop, the thermal effects in tissue are wavelength-dependent. From the infrared region (approx. 1.5 μm) towards the blue range, the absorption coefficient (*μ*_A_) and scattering coefficient (*μ*_S_) of the tissue is increasing.

At small laser outputs, the primary interaction of the radiation lies in the epithelium (Fig. 10). The epithelium consists of multi-layered cells without any kind of vessels (e.g., blood vessels) [35]. Therefore, no typical absorbers as melanin or hemoglobin are present for the spectral range investigated. If a non-initialized optical fiber is used, no tissue cutting effect was observed in the lower power range. This is shown in the higher cutting threshold values and can be clearly seen in Fig. 6 for 405 nm and 445 nm at 1 W in the non-contact mode. Here only thermal effects occurred based on the light absorption in the subjacent layers containing pigments and blood vessels. In the contact mode, incisions could be observed immediately at a laser output of, e.g., 1 W (Fig. 10). This cannot be explained by a scattering or absorption dependency due to the comparable incision depths at the wavelength range of between 400 and 1000 nm. Since all incisions were carried out with an initialized fiber tip, the cutting effect can be attributed to the purely heated fiber tip (hot tip). Upon increasing the laser power, the interactions occur in the blood-circulated subepithelial connective tissue. Here, fluctuations in the hemoglobin absorption, changes of the scattering properties of the tissue, and thermal tissue changes, e.g., by denaturation provide initial explanations that can be applied to the behavior of the efficiency factor.

The cut widths determined at all the wavelengths were far below the core diameter of the applied optical fiber. This can be attributed by optical effects regarding to the Gaussian beam distribution after the beam exit behind the fiber [36] as well as by the Gaussian beam distribution inside the tissue (aspect ratio *μ*_S_/*μ*_A_, [37]). Additionally, even if the attached gingiva is “significantly stiffer and more resistant to mechanical stresses than regions of non-keratinized oral mucosa” [38], a reduction of the incision width can be observed clinically caused by coagulation effects and as well as by the shrinking effect during tissue preparation [27].

### Results of efficiency—the efficiency factor *γ*_z_

In the literature, the “efficiency ranking” for the various diode lasers applied in the field of soft tissue surgery often leads to contradictory statements. This is essentially based on the parameter selection with respect to wavelength, operation mode, laser power settings, and the choice of the sample material and the handling (cut speed, angle of inclination). Within the scope of this study, for the first time, discernible relationships in the light-tissue interaction could be experimentally determined in a systematic and reproducible way due to the standardized experimental setup and regarding to the results by the determined statistical fluctuation range of the biological samples.

The resulting efficiency factor *γ*_z_ allows researchers to quantitatively assess the ratio of the fraction of the actual incision depth to the entire depth expansion of the thermal tissue interaction. In this regard, there have already been approaches in Fornaini et al., Goharkhay et al., and Janda et al., but which do not provide further in-depth evaluations [14, 39, 40]. In a retrospective study, Angiero et al. investigated the extent of thermal damage in oral biopsy specimens by using a diode laser (808 nm, CW, the power range of 1.6 W up to 2.7 W) [41]. The results show dimensional differences between the thermal damage zones of > 20%, which, for the power range of 1.6–2.7 W, is reflected in the corresponding efficiency factors of 0.51 up to 0.74 (Fig. 9).

### Clinical relevance

The quantitative classification of the efficiency factor can now be applied as a practice-relevant orientation value for laser incisions in the oral soft tissue of the gingiva. Especially important here is that a power increase does not absolutely affect to the same extent the incision depth and thermal tissue damage. Thus, the results of this current study refute the assumption of the null hypothesis.

**Fig. 12:**
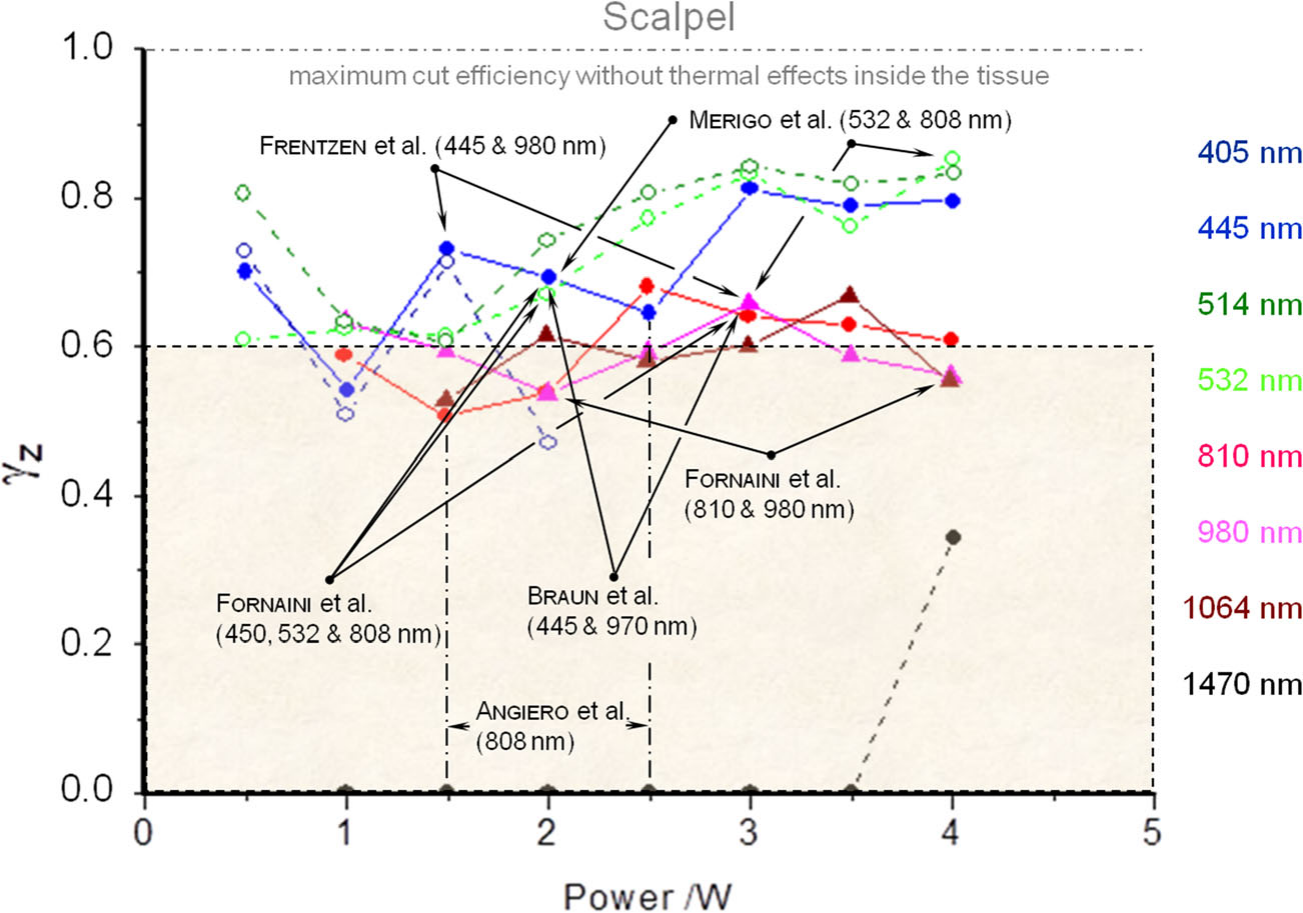
This diagram illustrates the relevance of the efficiency factor *γ*_z_ with respect to the literature [14, 29, 33, 34, 41, 42]. With the help of the defined threshold value *γ*_z_ = 0.6, the literature can now be quantitatively recorded and presented. Moreover, the height of the efficiency factor can be considered a clinical orientation value which can be transferred into practice

The efficiency factor *γ*_z_ of 0.6 was set as a clinical orientation value and was defined as the “lower threshold level”. For all wavelengths, the condition was there are at least two power values which show an efficiency factor at or above the value of 0.6. A confirmation of this parameter selection can be transferred from the literature into the model. This gives reference points about which laser parameters have proven to be successful in practice, and thus, lie on or even above an acceptable efficiency level. Figure 12 illustrates this relation. In the sense of a translational research, the results can be used to develop new treatment protocols, depending on the requirements of the clinician (cutting behavior, thermal influences).

The efficiency factor *γ*_z_ is not a factor that dictates whether a laser wavelength is “good” or “bad”. Instead, it allows forecasts concerning the controllability of the laser application on tissue. In this study, the best cut depth was attained at the wavelength 445 nm. In the power range of 0.5–2.5 W, however, certain fluctuations relating to the efficiency factor occurred. By contrast, a more uniform and, thus, predictable course in the cut efficiency with simultaneously good cutting performance were observed at the green wavelengths of 514 nm and 532 nm.

## Conclusion

To compare the influence of laser irradiation in the spectral range between 400 and 1500 nm, 8 wavelengths were exemplarily investigated on gingival tissue samples. The investigations have shown that power increases do not correlate to the same degree with incision depth and thermal tissue damage—a fact which impairs the controllability of a therapeutic measure. The efficiency factor *γ*_z_ describes those correlations and enables researchers to make forecasts about the expected tissue interaction with regard to cutting behavior and thermal effects as a predictor. Especially, thermal effects can be estimated and, as the result of future studies, may be exactly expressed numerically depending on the wavelength and tissue. This will accelerate the translational research in establishing novel laser wavelengths (e.g., 445 nm, 514 nm, and 532 nm), especially for dental applications.
